# Supplementary material for: Silver Nanoparticles In Situ Synthesized and Incorporated in Uniaxial and Core–Shell Electrospun Nanofibers to Inhibit Coronavirus
Source: Pharmaceutics. 2024 Feb 14;16(2):268. doi: 10.3390/pharmaceutics16020268 (PMC10893522; doi:10.3390/pharmaceutics16020268)
Supplement: Supplementary file 1 [file pharmaceutics-16-00268-s001.zip › pharmaceutics-2822995-supplementary.pdf]

SUPPLEMENTARY MATERIAL

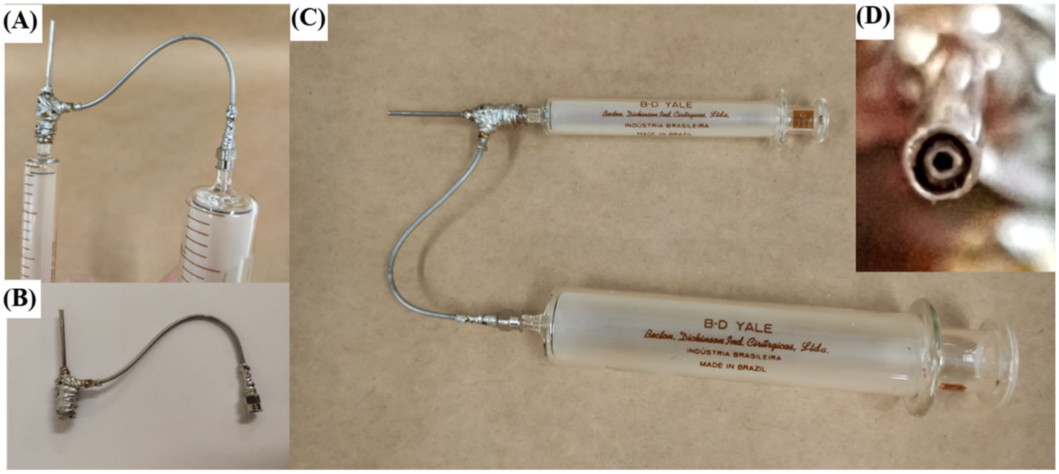

Figure S1. Prototype obtained for coaxial electrospinning (A-B) coaxial needles, (C) complete system simulating the application, and (D) centering of the needles. In the present study, the two syringes used were identical (10 mL).

Supplementary information for the virucidal assay methodology

For the in vitro virucidal assays, the culture medium for virus and cell line medium DMEM containing 10% fetal bovine serum (FBS) was used. Each of the nanofiber samples (PVA, PVA/AgNPs, PVA/CHT PVA/CHT/AgNPs, PCL[PVA/CHT], and PCL[PVA/CHT/AgNPs]) was placed in Petri dishes, under aseptic conditions, inside a class II biological safety cabinet and cut into equivalent size (50mm x 50mm). Then, 0.4 ml of DMEM (Gibco, USA) containing 40% Fetal Bovine Serum (FBS) containing the MHV-3 coronavirus (10^8 TCID50/ml), previously titrated, was added to the surface of each material and this test inoculum was then covered with a piece of film, which was gently pressed so that the inoculum spread uniformly across the face of the material and left for predetermined times (30', 1h, 8h, 24h, 48h, and 64h). EDTA Trypsin (Gibco, USA) was used to remove L929 cells adhered to the culture flask, allowing transfer of the cell suspension to 96 well plates Dulbecco's Minimal Essential Medium (DMEM; Gibco, USA) containing Fetal Bovine Serum (FBS) was used as a medium culture for the virus (2% FBS) and for cell line (10% FBS).

The aqueous dispersion of AgNPs were synthetized using 1x10^{-3} molL^{-1} of $AgNO_3$ as a silver precursor and 10x10^{-3} molL^{-1} of sodium citrate as a reducing and stabilizing agent. In this case, studies in healthy cells show a certain level of cytotoxicity, making it necessary to dilute the sample 1:10 (v/v) at 25 °C. This mixture was filtered using a sterile filter PTFE 0.45 µm.

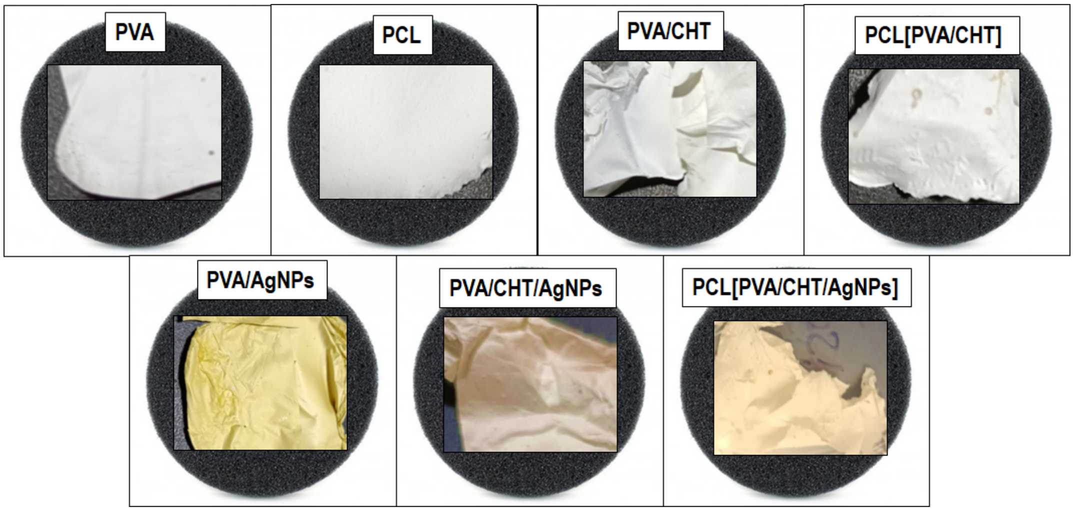

Figure S2. Nanofiber matrices obtained with (bottom) and without(above) AgNPs.

Table S1. Results are expressed as a percentage of viral inactivation compared to the untreated viral control [60].

Log's Reduction	Reduction Factor	Percentual of Inactivation/Reduction
1	10	90%
2	100	99%
3	1000	99.9%
4	10.000	99.99% VIRUCIDAL
5	100.000	99.999%
6	1.000.000	99.9999%
